# Cost-effectiveness of a rule-out algorithm of acute myocardial infarction in low-risk patients: emergency primary care versus hospital setting

**DOI:** 10.1186/s12913-022-08697-6

**Published:** 2022-10-21

**Authors:** Tonje R. Johannessen, Sigrun Halvorsen, Dan Atar, John Munkhaugen, Anne Kathrine Nore, Torbjørn Wisløff, Odd Martin Vallersnes

**Affiliations:** 1 Department of General Practice, Institute of Health and Society, University of Oslo, 1130 Blindern, 0318 Oslo, NO Norway; 2 Oslo Accident and Emergency Outpatient Clinic, City of Oslo Health Agency, Oslo, Norway; 3 Department of Cardiology, Oslo University Hospital Ullevaal, Oslo, Norway; 4 Institute of Clinical Medicine, University of Oslo, Oslo, Norway; 5 Department of Medicine, Drammen Hospital, Vestre Viken Hospital Trust, Drammen, Norway; 6 Department of Behavioural Medicine, Faculty of Medicine, University of Oslo, Oslo, Norway; 7 Health Services Research Unit, Akershus University Hospital, Lørenskog, Norway

**Keywords:** Chest pain, Troponin, Acute myocardial infarction, Acute coronary syndrome, Out-of-hours, Cost-effectiveness

## Abstract

**Aims:**

Hospital admissions of patients with chest pain considered as low risk for acute coronary syndrome contribute to increased costs and crowding in the emergency departments. This study aims to estimate the cost-effectiveness of assessing these patients in a primary care emergency setting, using the European Society of Cardiology (ESC) 0/1-h algorithm for high-sensitivity cardiac troponin T, compared to routine hospital management.

**Methods:**

A cost-effectiveness analysis was conducted. For the primary care estimates, costs and health care expenditure from the observational OUT-ACS (One-hoUr Troponin in a low-prevalence population of Acute Coronary Syndrome) study were compared with anonymous extracted administrative data on low-risk patients at a large general hospital in Norway. Patients discharged home after the hs-cTnT assessment were defined as low risk in the primary care cohort. In the hospital setting, the low-risk group comprised patients discharged with a non-specific chest pain diagnosis (ICD-10 codes R07.4 and Z03.5). Loss of health related to a potential increase in acute myocardial infarctions the following 30-days was estimated. The primary outcome measure was the costs per quality-adjusted life year (QALY) of applying the ESC 0/1-h algorithm in primary care. The secondary outcomes were health care costs and length of stay in the two settings.

**Results:**

Differences in costs comprise personnel and laboratory costs of applying the algorithm at primary care level (€192) and expenses related to ambulance transports and complete hospital costs for low-risk patients admitted to hospital (€1986). Additional diagnostic procedures were performed in 31.9% (181/567) of the low-risk hospital cohort. The estimated reduction in health care cost when using the 0/1-h algorithm outside of hospital was €1794 per low-risk patient, with a mean decrease in length of stay of 18.9 h. These numbers result in an average per-person QALY gain of 0.0005. Increased QALY and decreased costs indicate that the primary care approach is clearly cost-effective.

**Conclusion:**

Using the ESC 0/1-h algorithm in low-risk patients in emergency primary care appears to be cost-effective compared to standard hospital management, with an extensive reduction in costs and length of stay per patient.

**Supplementary Information:**

The online version contains supplementary material available at 10.1186/s12913-022-08697-6.

## Introduction

Primary care serves as a gatekeeper to the specialist healthcare system in order to reduce healthcare expenditure and unnecessary hospital admissions in many countries [1]. Chest pain and other symptoms suggestive of non-ST-segment elevation acute coronary syndrome (NSTE-ACS) represent a major challenge for primary care physicians due to a lack of sensitive diagnostic decision aids outside of hospital [2, 3]. Although the prevalence of acute myocardial infarction (AMI) in a primary care setting is usually below 5%, [4–6] diagnostic uncertainty results in defensive practice with increased hospital referral rates for the exclusion of an acute cardiac event [7–10]. Still, as demonstrated by Vester et al., more than 80% of these referrals end up with a non-cardiac diagnosis at discharge [11].

There is a growing international awareness to address issues related to overdiagnosis, [12–16] where extensive hospital admission of low-risk patients with chest pain and screening with high-sensitivity cardiac troponins (hs-cTn) are highlighted examples of overuse of care [14–16]. Studies from the Netherlands have shown that hospital admission of patients considered as false-positive ACS [6] or as low-risk by the HEART (History, Electrocardiogram (ECG), Age, Risk factors and Troponin) score [17] yield few additional health benefits despite substantial use of healthcare expenditure. Both studies further elaborated on the potential reduction in overall expenses if these low-risk groups were offered improved risk stratification outside the emergency departments (ED) [6, 17].

High efficacy, with subsequent reduction in costs, length of stay, and patient crowding in the EDs, has been demonstrated for patients triaged towards AMI rule-out by the European Society of Cardiology (ESC) 0/1-h algorithm for hs-cTn [18–21]. The 0/1-h algorithm was also listed as the preferred biomarker strategy in the *2020 ESC Guidelines for the management of acute coronary syndromes in patients presenting without persistent ST-segment elevation* [22]. In previous work from the observational OUT-ACS study (*One-hoUr Troponin in a low-prevalence population of Acute Coronary Syndrome*), [23] we demonstrated a high rule-out safety for AMI (sensitivity 98.4%, negative predictive value 99.9%) by using the ESC 0/1-h algorithm for hs-cTnT in an emergency primary care setting. In addition, 80.5% of the patients were conclusively triaged by the algorithm, and only 13.2% of 1711 patients required hospitalisation [23]. With these results in mind, we hypothesise that using the 0/1-h algorithm outside the hospital EDs would substantially reduce additional advanced testing, unnecessary hospitalisations, and overall expenses. To the best of our knowledge, the potential reductions in health care expenditure saved by applying the ESC 0/1-h algorithm in emergency primary care have so far not been studied.

### Objectives

This study aimed to explore the cost-effectiveness of assessing low-risk patients with chest pain using the ESC 0/1-h algorithm for hs-cTnT in emergency primary care compared to routine hospital management. In addition, the differences in direct costs and length of stay per low-risk patient between the two settings were investigated.

## Material and methods

### Study design

In this cost-effectiveness analysis, we compared a cohort of patients considered low-risk for NSTE-ACS and managed with the 0/1-h algorithm in emergency primary care to a comparable low-risk hospital cohort. Data from the prospective, observational OUT-ACS study, [23] conducted at the Oslo Accident and Emergency Outpatient Clinic (OAEOC) from November 2016 to October 2018, were used to calculate direct costs and additional length-of-stay for the emergency primary care setting. These estimates were compared with patients considered low risk for NSTE-ACS at Drammen Hospital in 2018. The chosen analytical method combines empirical data from the OUT-ACS study and a simulation model.

### Study settings and locations

The OAEOC is the main primary care emergency clinic in Oslo, Norway, which serves the entire city of Oslo 24/7 all year, with approximately 200 000 consultations annually. Unlike most Norwegian out-of-hours (OOH) clinics, the clinic has available chest x-ray service, facilities for observation of patients for up to 24 h, and a possibility of having venous blood samples sent to hospital for analysis. Otherwise, the OAEOC is a standard primary care emergency clinic with limited diagnostic and therapeutic options, staffed by general practitioners (GPs) and nurses.

Drammen Hospital was chosen as the comparator to the OAEOC, as the primary care emergency clinics in the region of Drammen do not offer hs-cTn measurements. Hence, all patients in need of a safe exclusion of AMI are hospitalised. Drammen Hospital is a large general hospital in Vestre Viken Hospital Trust, with a total catchment area of 168 000 inhabitants [24].

### Clinical assessment of low-risk patients

**Fig. 1:**
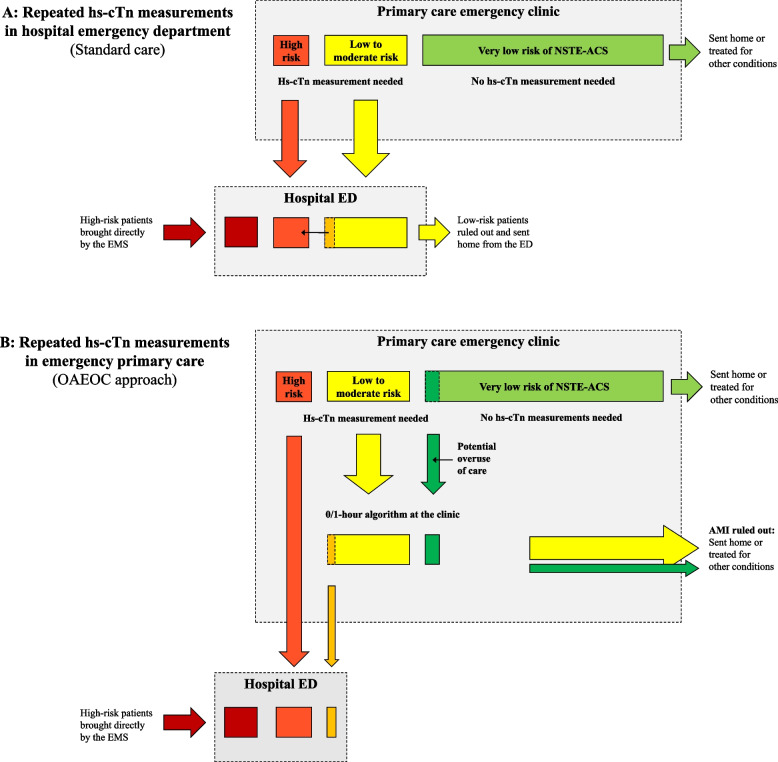
The different management strategies of low-risk patients with chest pain at hospital versus emergency primary care. The estimated reduction in health care utilisation by initially assessing the low-risk group outside of hospital ED is visualised by the missing yellow square at hospital level in Fig. 1B. AMI: acute myocardial infarction; ED: emergency department; EMS: emergency medical services; hs-cTn: high-sensitivity cardiac troponin; NSTE-ACS: non-ST-segment elevation acute coronary syndrome; OAEOC: Oslo Accident and Emergency Outpatient Clinic

In Norway, all patients with acute chest pain are advised to call the emergency services. As the vast majority will have an ambulance dispatched, patients with STEMI or patients considered critically ill generally bypass primary care. All others are initially assessed in primary care, either by their regular GP during office hours or by GPs at out-of-hours/primary care emergency clinics. In most cases, standard chest pain assessment comprises medical history, focused clinical examination, vital signs, and a 12-lead ECG. If an ACS is suspected or cardiac troponins are considered necessary to exclude an AMI, the patient is transferred to a hospital. This is also the setting in Drammen (Fig. 1A and Online Figure S1; standard care). Some GPs do have access to prehospital point-of-care troponin assays, but these assays do currently not provide adequate sensitivity for a safe AMI rule-out [3, 25, 26].

At the OAEOC, a third diagnostic option has been available since 2009. Patients considered clinically stable with resolved chest pain and without urgent need for hospital transfer may be offered serial hs-cTnT measurements at the clinic. This group comprises patients with resolved chest pain with either an increased cardiovascular risk profile, non-specific findings at the ECG of unknown clinical relevance, or patients with atypical symptom presentation (acute fatigue, dyspnoea, or diaphoresis). While these patients wait at the clinic, the blood samples are sent to the central laboratory at Oslo University Hospital Ullevaal by courier transport (approximately 4 kms) at a 4-h interval. An additional 1-h hs-cTnT measurement was sampled during the OUT-ACS study [23]. If the hs-cTnT routine had not been available at the clinic, most of these patients would have been transferred to hospital.

At Drammen Hospital ED, a complete clinical examination, repeated ECGs, a standard blood test panel, and a chest x-ray are obtained from all patients admitted with chest pain or other symptoms suggestive of NSTE-ACS. Additional diagnostic workup and treatment are offered if considered necessary by the treating physician. The ESC 0/1-h algorithm was not implemented at Drammen hospital, and hs-cTnI was measured at admission and approximately six hours later. The non-specific ICD-10 (*International Statistical Classification of Diseases and Related Health Problems, 10th Revision*) [27] codes R07.4 (*chest pain, unspecified*) or Z03.5 (*observation for other suspected cardiovascular diseases*) are set in the absence of an elevated cardiac troponin or a more specific diagnosis at discharge. Patients registered with R07.4 and Z03.5 would most likely be triaged as rule-out and discharged home if the 0/1-h algorithm had been available at the emergency primary care level in Drammen. In the following analyses, we consider the low-risk hospital cohort comparable to the group offered hs-cTnT measurements at the OAEOC. Details regarding the ESC 0/1-h algorithm are described in Online Figure S2, and the two management strategies and levels of care are illustrated in Fig. 1 and Online Figure S1.

### Outcome measures

The primary outcome measure was the costs per quality-adjusted life-year (QALY) of applying the ESC 0/1-h algorithm in emergency primary care compared to routine hospital management. The secondary outcome measures were the estimated healthcare cost and length of stay per patient in the two settings.

For estimation of QALY, potential health loss due to the estimated length of stay was multiplied by estimates of health-related quality of life among patients with AMI, [28] when considering the potential of a minimal increase in AMIs in the primary care cohort. The OUT-ACS study reported 0.2% AMIs (2/1232) the following 30 days among those ruled out by the algorithm and discharged home (One AMI at index and another on day 5), and three events (2 AMIs; 1 death) among the non-hospitalised patients in the observation group (*n* = 243) [23]. Therefore, total events after 30-days were 0.3% (5/1485) (Table 1), which was applied to estimate the lifetime health-related quality of life lost by not being hospitalised. As the low-risk hospital cohort comprises administrative data only, we applied numbers from a comparable Norwegian hospital cohort to assess the 30-day event rate at hospital level. In the 2019 publication by Bjørnsen et al., there were two events (1 death; 1 ACS) the following 30 days among 862 patients discharged with a non-specific chest pain diagnosis (R07.4) [29]. In addition to the 0.2% incidence rate, [29] we assumed an average age of 56 years and quality of life weights as reported by Wisløff et al. [28] and in official Norwegian guidelines for economic evaluations [30].

**Table 1:** Baseline characteristics of the low-risk group at the primary care emergency clinic

	**OUT-ACS** **total** ***n* = 1711** **(100%)**	**Not admitted to hospital** ***n* = 1485** **(86.8%)**	**Admitted to hospital** ***n* = 226** **(13.2%)**
**Male sex, n (%)**	895 (52.3)	764 (51.4)	131 (58.0)
**Age, median (IQR)**	56 (45–68)	55 (44–66)	63.5 (51–73)
**Risk factors for CVD, n (%)**			
Current/history of smoking	449 (26.2)	387 (26.1)	62 (27.4)
Previous coronary artery disease	317 (18.5)	262 (17.6)	55 (24.3)
Hypertension	448 (26.2)	379 (25.5)	69 (30.5)
Dyslipidaemia	422 (24.7)	369 (24.8)	53 (23.5)
Other CVD^a^	288 (16.8)	228 (15.4)	60 (26.5)
Diabetes mellitus	171 (10.0)	143 (9.6)	28 (12.4)
COPD	80 (4.7)	58 (3.9)	22 (9.7)
Family history of CVD	691 (40.4)	603 (40.6)	87 (38.5)
**Presenting acute symptoms (%)**			
Chest pain	1486 (86.8)	1301 (87.6)	184 (81.4)
Constricting	1239 (72.4)	1082 (72.9)	157 (69.5)
Sharp	404 (23.6)	358 (24.1)	46 (20.4)
Tearing	64 (3.7)	58 (3.9)	6 (2.7)
Burning	208 (12.2)	183 (12.3)	25 (11.1)
Respiratory dependent	302 (17.7)	251 (16.9)	51 (22.6)
Chest wall tenderness	205 (12.0)	184 (12.4)	21 (9.3)
Movement dependent	219 (12.8)	197 (13.3)	21 (9.3)
Other pain (abdomen, back, neck)	48 (2.8)	39 (2.6)	9 (4.0)
No pain	177 (10.3)	144 (9.7)	33 (14.6)
Pain radiation	972 (56.8)	865 (58.2)	135 (59.7)
Dyspnea	901 (52.7)	768 (51.7)	133 (58.8)
Palpitations	637 (37.2)	558 (37.6)	79 (35.0)
Syncope/pre-syncope	460 (26.9)	391 (26.3)	69 (30.5)
Acute fatigue	571 (33.4)	488 (32.9)	83 (36.7)
Nausea and/or vomiting	732 (42.8)	641 (43.2)	91 (40.3)
Diaphoresis	561 (32.8)	490 (33.0)	71 (31.4)
**First ECG, n (%)**			
Normal	1515 (88.5)	1332 (89.7)	183 (81.0)
Non-specific changes^b^	196 (11.5)	153 (10.3)	43 (19.0)
**Symptom onset to first hs-cTnT, n (%)**			
< 3 h	182 (10.6)	161 (10.8)	21 (9.3)
3 – 5.99 h	609 (35.6)	532 (35.8)	77 (34.1)
6 – 11.99 h	409 (23.9)	336 (22.6)	73 (32.3)
> 12 h	511 (29.9)	456 (30.7)	55 (24.3)
**According to the 0/1-h algorithm**			
Rule-out (0/1 h)	1311 (76.6)	1232 (83.0)	79 (35.0)
Observation group (0/1 h)	334 (20.5)	243 (16.4)	91 (40.3)
Rule-in (0/1 h)	66 (3.9)	10 (0.7)	56 (24.8)
**HEART risk score**			
Low risk (0–3 points)	871 (50.9)	805 (54.2)	66 (29.2)
Intermediate risk (4–6 points)	760 (44.4)	633 (42.6)	127 (56.2)
High risk (7–10 points)	80 (4.7)	47 (3.2)	33 (14.6)
**Endpoints**			
Myocardial infarctions at index	61 (3.6)	1 (0.1)	60 (26.5)
Myocardial infarctions after 30 days	3 (0.2)	3 (0.2)	0 (0.0)
Myocardial infarctions after 90 days	2 (0.1)	1 (0.1)	1 (0.4)
Deaths after 30 days	5 (0.3)	1 (0.1)	4 (1.8)
Deaths after 90 days	4 (0.2)	1 (0.1)	3 (1.3)

All values are presented as n (%) or median (IQR). As the low-risk hospital cohort was obtained from administrative data only, we do not have additional baseline characteristics for these patients. However, for the purpose of this analysis, we consider the non-hospitalised OUT-ACS cohort comparable to the low-risk patients at Drammen hospital

*COPD* Chronic obstructive pulmonary disease, *CVD* Cardiovascular disease, *ECG* Electrocardiogram, *hs-cTnT* High-sensitivity cardiac troponin T, *IQR* Interquartile range; One hoUr Troponin in a low-prevalence of Acute Coronary Syndrome

^a^Includes atrial fibrillation, other arrhythmias, cardiomyopathies, cerebral stroke, heart failure, or valvular disease

^b^Non-specific changes in either the ST-segment, T-inversions, Q-waves, atrial fibrillation, pacemaker or left/right bundle branch block of unknown clinical significance

### Estimating healthcare resources

Initial resources spent in emergency primary care, comprising patient registration, triage, clinical examination, and ECG, were assumed similar in Oslo and Drammen regardless of the availability of hs-cTn in primary care. Similar assumptions apply to costs related to service, administration, buildings, and the initial use of emergency medical services (EMS) to the primary care emergency clinics. Cost estimates at the OAEOC also comprise hs-cTnT measurements, additional diagnostic tests and procedures, personnel resources applied per patient assessed by the 0/1-h algorithm and potential referrals to outpatient cardiac testing after OAEOC discharge. Personnel resources (minutes spent per patient) were estimated by consulting experienced senior personnel at the OAEOC. Data on the probabilities of using a specific test or procedure was calculated by investigating patient records from a random selection of the OUT-ACS study cohort (*n* = 171 of 1711; 10%). We also assume that making hs-cTnT measurements available in primary care would lead to some overuse of care (estimated 10–15%) with more patients made subject to triage by the algorithm. These patients were already part of the OUT-ACS cohort (Fig. 1B, dark green).

For the hospital setting, anonymous, aggregated data from Drammen hospital were extracted from the hospital records for all patients discharged with a final non-specific cardiac diagnosis (R07.4 and Z03.5) from January to December 2018. Patients with elevated hs-cTn measurements were not part of the low-risk hospital cohort, as these patients most likely would have been discharged with a more specific diagnosis (e.g., heart failure, atherosclerotic heart disease, arrhythmias, cardiomyopathies, peri-myocarditis, pulmonary embolism, or sepsis). The variables extracted were age, sex, length of stay, procedure codes and Diagnosis-Related Group (DRG) codes. DRG is a patient classification system that standardises all charges associated with an inpatient stay from admission to discharge [30, 31]. Experienced senior personnel were consulted to estimate the use of additional diagnostic tests and procedures not encompassed by the procedure codes.

### Estimating costs

All costs were based on 2020 averages and fees (2020 EUR 1.00 = NOK 10.73). According to Norwegian guidelines, prices and health estimates during future years were discounted at a 4% discount rate [30].

At the OAEOC, average personnel costs (per hour) were delivered by the finance consultant at the City of Oslo Health Agency. Chest x-ray and venous blood samples were calculated as outpatient radiological and laboratory services. According to *The Norwegian Medicines Agency’s Guidelines for the submission of documentation for single technology assessment of pharmaceuticals,* [30] the costs of the personnel used were based on average pay multiplied by 1.3 to include payroll taxes and other social charges. Hospital services were estimated as if financed by full reimbursements from *The Norwegian Health Economics Administration* (HELFO). Services provided by GPs and primary care emergency clinics were calculated by multiplying the HELFO reimbursements by two to cover other financing sources. Outpatient radiological and laboratory services were estimated as the reimbursed sum from HELFO plus the fee paid by the patient, multiplied by two, to include personnel costs at the radiology and lab units [30]. The estimated reduction in low-risk ED admissions with the 0/1-h algorithm at the primary care emergency clinic is visualised by the missing yellow square at hospital level in Fig. 1B.

At Drammen Hospital, the overall costs were based on the reported DRG codes for the low-risk cohort. In addition, the total number of diagnostic tests, procedures, and length of stay were reported separately. Estimated mean costs related to the use of ambulances, including personnel and equipment, were reported by the Prehospital Division at Oslo University Hospital, Ullevaal.

### Analytical methods

The health economic evaluation was performed using a decision-analytic model incorporating a simple Markov model taking long-term differences between interventions into account [32]. The structure of the decision model is illustrated in Online Figure S1. The analysis included the Markov model to provide long-term insights into impacts beyond the first year after presenting with ACS symptoms. To include potential differences in rates of ACS, the model was constructed to consist of the three health states: non-CVD, CVD and dead. One-year cycle length was chosen, and a half-cycle correction was applied to account for events occurring on average halfway through cycles. Living with CVD was assumed to have a hazard ratio of 1.6 compared to living without, based on two Norwegian analyses [33, 34]. Details on other inputs are included in the Appendix.

In addition to a base case (i.e., most likely) model, separate analyses were conducted to evaluate a conservative scenario. Four inputs were chosen, not based on what is considered most likely, but as a worst-case scenario for managing these patients in primary care. These were: 1) Costs of time spent on the 0/1-h algorithm based on tariffs instead of personnel wages as reported in Online Table S1. 2) Incorporating a potential increase in AMIs at the OAEOC, as reported under outcome measures. 3) Costs related to a lower probability of ambulance transport for hospital admissions from primary care in Drammen. 4) Additional length of stay at the OAEOC, where the upper range of uncertainty was selected as the estimate. Probabilistic sensitivity analysis of the parameters in the base case model was also conducted and presented in the Supplementary appendix and Figures S2 and S3.

Current Norwegian assumptions regarding the threshold for cost-effectiveness are cited to be between Norwegian Kroner (NOK 275,000 and 825,000 per QALY, i.e., between Euro (EUR) 25,600 and EUR 76,900 per QALY) [35].

## Results

### Baseline description of the low-risk patients

Baseline characteristics of patients from the OUT-ACS study not being hospitalised using the 0/1-h algorithm (*n* = 1485, 86.8%) are described in Table 1. The median age was 55 (IQR 44–66) years, and 51.4% were males. The low-risk patients admitted to Drammen hospital (*n* = 567) had a median age of 57 (IQR 46–69) years, and 54.3% were males.

### Estimated health care expenditure

The additional costs of implementing the ESC 0/1-h algorithm at the primary care emergency clinic were estimated to be either EUR 230 or EUR 192 for each low-risk patient in need of hs-cTnT measurements. DRG tariffs are not used for cost calculations in primary care. The estimate, therefore, comprises direct costs of laboratory and additional procedures (EUR 41), personnel costs, either by tariffs (EUR 137) or by wages (EUR 99), and estimated costs related to increased referrals to outpatient cardiac testing (EUR 52) (Table 2 and Table S1-2). The estimated reduction in health care expenditure for each low-risk patient assessable by the 0/1-h algorithm outside of hospital was EUR -1672 per patient with the most conservative scenario and EUR -1794 with the base case scenario (Table 2).

**Table 2:** Assessment of low-risk patients with chest pain in the two settings

	**0/1-h algorithm at emergency primary care** *OUT-ACS cohort, Oslo (n* = *1485)*		**All hs-cTn measurements at hospital ED** *Low-risk cohort, Drammen (n* = *567)*		**Differences**
	**Conservative scenario**	**Base case scenario**	**Conservative scenario**	**Base case scenario**	
**EMS to emergency primary care** *(Costs per transport)*	**€ 162** *(€ 559;* *29%)*	**€ 162** *(€ 559;* *29%)*	**€ 162** *(€ 559;* *29%)*	**€ 162** *(€ 559;* *29%)*	**€ 0** *(assumed similar)*
**Primary care emergency clinic** *General costs/ consultation*^a^	**€ 166**	**€ 166**	**€ 166**	**€ 166**	**€ 0** *(assumed similar)*
*Additional costs with a 0/1-h algorithm*	**€ 230** • *Diagnostics € 41* • *Personnel, tariffs € 137* • *Cardiac outpatient testing € 52*	**€ 192** • *Diagnostics € 41* • *Personnel, wages € 99* • *Cardiac outpatient testing € 52*	**(none)**	**(none)**	**€ 230 *or* 192**
**EMS to hospital** *(costs per transport)*	**(none)**	**(none)**	**€ 419** *(€ 559;* *75%)*	**€ 503** *(€ 559;* *90%)*	**€ -419 *or* -503**
**Hospital** (*DRG tariffs*^a^*)*	**(none)**	**(none)**	**€ 1483**	**€ 1483**	**€ -1483**
**TOTAL**	**€ 558**	**€ 520**	**€ 2230**	**€ 2314**	**€ -1672 *or* -1794**
**LOS**	**Mean: 4.0 h**	**Mean: 3.4 h**	**Mean: 22.3 h**	**Mean: 22.3 h**	**-18.3 h *or* -18.9 h**
**QALY**	**-0.00760** *LOS: -0.00011* *AMI: -0.00749*	**-0.00009** *LOS: -0.00009* *AMI: -0.0*	**-0.00574** *LOS: -0.00059* *AMI: -0.00515*	**-0.00059** *LOS: -0.00059* *AMI: -0.0*	**-0.00186 *or* + 0.00050**

Details regarding cost estimates, probabilities and calculations are listed in Online Tables S1, S2, and S4. All numbers are adjusted to 2020 figures

*AMI* Acute myocardial infarction, *DRG* Diagnosis-related groups, *EMS* Emergency medical services, *ED* Emergency department, *EUR* Euro, *LOS* Length of stay, *OUT-ACS* One-hoUr Troponin in a low-prevalence population of Acute Coronary Syndrome, *QALY* Quality-adjusted life year

^a^General costs by standard consultation per patient encompass service costs, building, personnel, administration etc, assumed similar at the primary care emergency clinics in Oslo and Drammen

For the low-risk cohort (*n* = 567) at Drammen hospital, the total DRG was calculated to EUR 840,664, with a mean cost for one low-risk patient of EUR 1483 (Table 2 and Online Table S1). ECG, standard blood panel (Online Table S1) and chest x-ray were obtained from all patients on admission. Additional advanced procedures (e.g., stress ECG and echocardiogram), were performed in 31.9% (*n* = 181) of the low-risk group (Online Table S3). In addition, following standard prehospital routine, most patients hospitalised with chest pain suggestive of ACS are transported from emergency primary care by ambulance, with an estimated cost per transport of EUR 559 (Online Table S1).

### Length of stay

In the base case scenario, the additional length of stay at the OAEOC, using the 0/1-h algorithm, and at Drammen Hospital were 3.4 h (SD 0.740) and 22.3 h (SD 22.010), respectively. In the conservative scenario, the upper range of uncertainty was chosen for the mean additional length of stay at the OAEOC, at 4.0 h (SD 0.870). Subsequently, the mean difference in length of stay between the two settings was -18.9 h in the base case scenario and -18.3 h in the more conservative scenario (Table 2 and Online Table S4).

### Base case cost-effectiveness

In our base case analysis, QALY loss related to length of stay was 0.00009 at the OAEOC and 0.00059 at Drammen Hospital, leading to 0.00050 lower QALY with standard hospital treatment than at the OAEOC (Table 2). As the 30-day event rates in both low-risk cohorts were below the potentially acceptable AMI miss rate of ≤ 1%, [10, 36] the health loss due to missed events was estimated at 0.0 in our base case scenario in both settings. With increased health due to less waiting time and decreased costs per patient (EUR -1794), the OAEOC strategy is cost-effective regardless of the cost-effectiveness threshold, commonly referred to as a *dominant* strategy in health economics.

### Conservative scenario

**Fig. 2:**
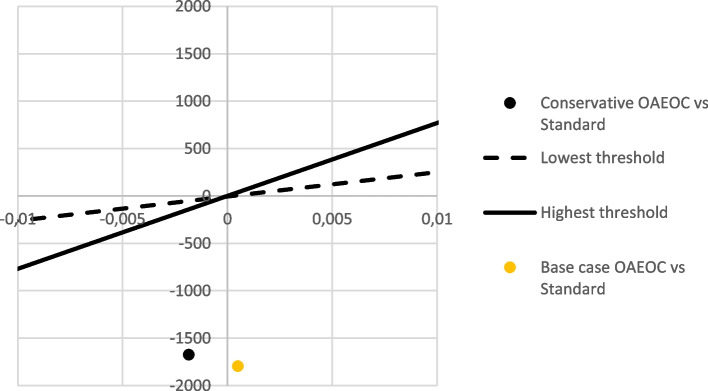
Cost-effectiveness of emergency primary care versus standard hospital management. The graph illustrates the difference in health on the x-axis and in costs on the y-axis. The lines through the graph indicate the suggested minimum and maximum cost-effectiveness thresholds (cited to be between EUR 25,600 and EUR 76,900 per QALY) for Norway [35]. The health lost due to missed AMIs at the primary care level will be bigger than the health gained by less waiting in hospital, as indicated by the negative health on the graph. Still, with a difference of EUR -1672 or -1794 per patient, the estimated QALY is well below the current assumed threshold for cost-effectiveness in Norway, implying that the primary care approach is cost-effective. OAEOC: Oslo Accident and Emergency Outpatient Clinic; QALY: quality-adjusted life years

Among the non-hospitalised patients in the OUT-ACS cohort, the 30-day combined incidence rate for AMI and deaths was 0.3%. The rate was assumed to be similar to Bjørnsen et al. at 0.2% for the hospital setting [27] and included in our conservative scenario. Estimated discounted remaining QALYs for an average person at 56 years old was estimated at 13.3 QALYs, while for a person who had experienced an AMI mounted to 11.1 QALYs. An assumed increased AMI rate of 0.1% at the OAEOC compared to the hospital would result in an additional 0.0023 QALYs lost. Including QALYs saved due to shorter length of stay, health loss in the conservative scenario is reduced to -0.0019 QALYs with the algorithm at the OAEOC. With a reduction of EUR 1672, the cost per QALY lost equals EUR -1672 / -0.0019 QALYs = EUR 880 000 per QALY. As can be seen from Fig. 2, this is well below the currently assumed thresholds for cost-effectiveness in Norway, implying that the OAEOC is cost-effective in Norway also in the conservative scenario.

### Potential generalisability

**Fig. 3:**
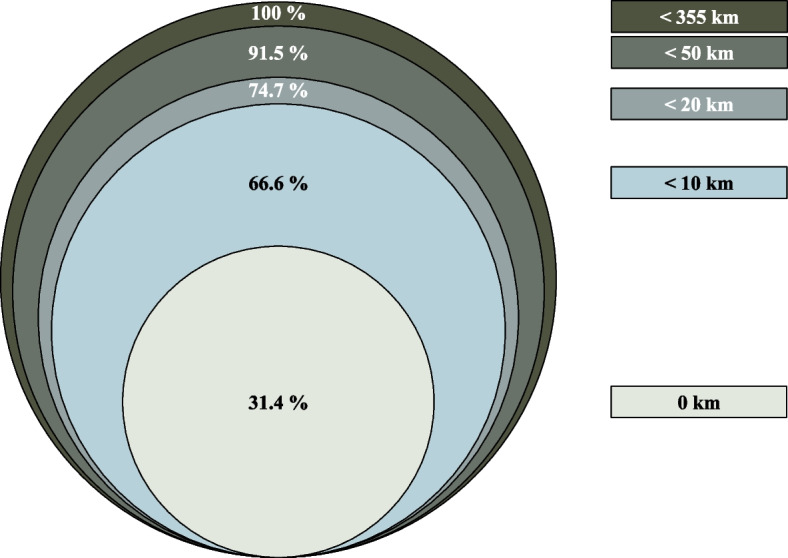
Proportions of the Norwegian population (*n* = 5,367,580 in 2020) with emergency primary care/out-of-hours clinic located within the specified distance from the nearest hospital with an available hs-cTn assay. Km: kilometres; hs-cTn: high-sensitivity cardiac troponin

Thirty-two of the total 169 Norwegian OOH-/primary care emergency clinics, with a catchment area population of 1.7 million (31.4% of the Norwegian population), are located on hospital grounds, enabling optimal use of the 0/1-h algorithm if implemented in routine clinical care [37]. As an example, the catchment area population expands to 4.0 million (74.7%) if the acceptable distance to an available hs-cTn lab is set to 20 km (with a mean courier drive of 11.1 min) (Fig. 3 and Online Table S5). In 2014, 16,320 patients were discharged from Norwegian hospitals with the non-specific ICD-10 code R07 (*pain in throat and chest*), the second most common diagnosis following an acute somatic hospital admission [38, 39]. Among them, 7613 were referred after an OOH assessment [39]. Based on our figures, if all patients with an OOH clinic located within 20 kms of an available lab (74.7%; *n* = 5687) were assessed at the clinic with the 0/1-h algorithm, 13.2% would be hospitalised (*n* = 751), and 86.8% (*n* = 4936) would be discharged home (Table 1). The following cost reduction per low-risk patient of EUR 1672 to 1794 would result in an estimated reduction of EUR 8.3 to 8.6 million per year in Norway. This number is potentially larger as 3923 of the R07 admissions were directly hospitalised by the ambulance [39]. We have reason to believe that some of these would have been brought to an OOH clinic in case of available hs-cTn assessment.

## Discussion

This cost-effectiveness analysis documents the potential benefits and cost reductions if the initial assessment of low-risk patients with chest pain, using the ESC 0/1-h algorithm, is performed outside of the hospitals. This indicates that introducing the algorithm in emergency primary care would set free health care resources that would gain more health elsewhere than health loss due to a potential minimal increase in AMIs. A considerable potential reduction in healthcare costs, estimated to EUR -1672 to EUR -1794 per low-risk patient, was demonstrated when serial hs-cTn measurements were offered at the primary care level rather than in a hospital ED. In addition, the total length of stay would be reduced from 22.3 h to between 3.4 and 4.0 h by using the 0/1-h algorithm in emergency primary care compared to traditional hospital assessment.

Comparable numbers of ED admissions of low-risk patients with chest pain (R07.4; 876 per 300 000 inhabitants) and hospital LOS (median 22 h) were documented by Bjørnsen et al. [29]. In addition, similar costs estimates per low-risk admission, i.e. EUR 1448 [6], EUR 1360, [11] and EUR 1580, [17] have been reported in recent studies from the Netherlands.

Implementation of the 0/1-h algorithm for hs-cTn in primary care requires a short distance to an available hs-cTn assay 24/7. In Norway, this is mainly restricted to hospital EDs. As significant geographical variations exist between urban and rural districts in Norway, we acknowledge that broad implementation of the algorithm is not feasible. In Norway, 32% of patients admitted with non-specific chest pain (R07) and 50% of all AMIs (K21) bypass primary care by being directly hospitalised by the ambulance service, especially in central areas [38, 39]. If the 0/1-h algorithm were implemented in primary care, hospitalisation of low-risk patients is expected to be reduced in central areas with a short distance to an ED. Like in Norway, several European countries have merged smaller OOH clinics into larger cooperations with increased catchment areas and more centralised locations [40]. In 2014, 63% of the OOH services in the Netherlands were located adjacent to a hospital ED but without more extensive access to diagnostics tests or troponins [41]. Therefore, the 0/1-h algorithm approach for the primary care setting might also be transferable to other countries with a similar organisation model.

A study by Mokhtari et al. found that the performance of the 0/1-h algorithm combined with interpretation of the ECG and medical history-taking did not change by the physician's experience [42]. Hence, implementing such an algorithm should also be feasible and user-friendly for GPs on OOH rotation.

One of the main decisions made by primary care physicians is whether a patient needs to be directly hospitalised or further assessed in primary care [1]. The fear of missed AMIs would probably result in some overuse of hs-cTn measurements at the primary care level to support the decision process. At the OAEOC, overuse of hs-cTnT measurements is estimated to 10–15% by experienced senior GPs (illustrated by the dark green area in Fig. 1B). These 15% represent patients who most likely would have been discharged home without further testing at the primary care emergency clinic in Drammen. Implementing a diagnostic test in a low-prevalence setting may also contribute to more false-positive results and unnecessary hospitalisations. In the OUT-ACS study, the rule-in group had a specificity of 98.7% and a sensitivity of 73.8% [23]. Among 1000 patients with a 3.6% AMI prevalence, 36 patients would have an AMI, and 13 patients a false-positive test in the rule-in group. Still, most patients transferred to the hospital with a false positive hs-cTnT were admitted with other acute conditions requiring a higher level of care (e.g., acute heart failure, pulmonary embolism, or peri-myocarditis). Simultaneously, none of the false positives discharged home suffered an AMI or died the following 90-days [23]. We, therefore, conclude that assessment with the 0/1-h algorithm in emergency primary care is sufficient for the low-risk group. This strategy is consistent with the comprehensive gatekeeper function of primary care, which is to offer patients appropriate and adequate healthcare at the lowest effective level [1, 43]. Also, by not offering hs-cTn measurements at the OAEOC, a substantial proportion of the non-hospitalised patients (*n* = 1485; Table 1) would probably have been directly hospitalised at substantially higher costs.

### Limitations

Some limitations merit consideration: First, only the theoretical cost-effectiveness of assessing low-risk chest pain outside of hospital is investigated in this analysis. The study is based on data from the observational OUT-ACS cohort and not a real-world implementation study, which would be preferable.

Second, in this economic evaluation analysis, we cannot ensure that the assessment of low-risk patients with chest pain at the primary care level is comparable to hospital. However, the 30-day event rate in the non-hospitalised OUT-ACS cohort (Table 1) is similar to the low rate found among low-risk patients at a large Norwegian hospital [29]. Two of the four AMIs in the OUT-ACS cohort the following 30 days were assigned to the observation group by the 0/1-h algorithm. Improved recommendations [22] and recently validated novel criteria for patients in the observation group [44, 45] are expected to enhance the assessment of patients in the observation group.

Third, the estimates for the hospital arm come from a single hospital, which may limit the generalisability of our findings. However, the catchment area of Drammen hospital is reasonably representative of the Norwegian population regarding age distribution, morbidity, and mortality [46]. Furthermore, there are only minor differences between Drammen hospital and Norway in general regarding important quality indicators such as 30-day mortality rates, 30-day CVD mortality rates and risk of hospital readmission. Also, standardised tools for prioritising patients at the primary care emergency department in Drammen are similar to national data. Thus, it seems likely that there are only minor differences in the population and clinical management at Drammen compared to the rest of Norway.

Fourth, several of our estimates are based on best guesses and uncertain assumptions. For this reason, a base case and a conservative scenario were estimated (Table 2). For the low-risk hospital cohort, only ICD-10 R07.4 and Z03.5 were extracted from the administrative database. There were probably additional low-risk patients at Drammen hospital, discharged with a more specific ICD-10 diagnosis (e.g., anxiety disorder, gastritis, or myalgia).

Fifth, even though the 2020 ESC guidelines recommend the 0/1-h algorithm, [22] the algorithm is still not implemented at Drammen hospital. However, in a before-after-cohort from six EDs in Sweden, in-hospital length of stay and costs per patient were reduced to 4.7 h and $1079 (= EUR 927) after implementing a rule-out strategy combining the ESC 0/1-h algorithm and the HEART score [21]. Similar reductions would be expected for the low-risk hospital cohort in Drammen in case of implementation. Still, the additional costs of applying the algorithm in primary care will be lower (EUR 192; Table 2).

Sixth, the calculation of potential budget impact is based on a Norwegian registry on acute somatic hospital admissions in 2014, which reports numbers on the ICD-10 R07 group combined and not the R074 separately [39]. In the calculation, we also assume that the national R07 admissions were distributed equally across all the Norwegian OOH-clinics according to geographical location, which will not be the case in a real-world setting. Still, we believe the calculation may contribute to visualising potential cost reduction provided by the algorithm outside of the EDs.

Finally, implementing the 0/1-h algorithm for hs-cTn in primary care requires a short distance to an available lab and a similar healthcare organisation model, including a gatekeeper function in primary care and referral-based access to the ED. Nevertheless, there is increased support for an initial assessment of patients considered as low risk at a lower level of care [6, 17]. A study from the ED setting recently concluded that additional diagnostic procedures (e.g., stress test, echocardiography and coronary angiography) for patients triaged as rule-out by the algorithm had few diagnostic benefits and more false positives [47]. Hence, implementing the algorithm for assessing low-risk patients in emergency primary care could potentially result in less advanced testing, as these procedures are not available at the primary care level.

Newly developed hs-point-of-care assays for troponins have shown comparable diagnostic performance as central lab assays [48, 49]. If these could be integrated within a 0/1-h algorithm for the primary care setting in the future, broader implementation and enhanced diagnostic chest pain assessment outside of hospital might also be possible in rural areas.

## Conclusion

Assessment of acute chest pain in patients considered low risk of NSTE-ACS, using the ESC 0/1-h algorithm in emergency primary care, appears to be cost-effective compared to routine hospital management. This approach may significantly reduce healthcare costs, length of stay and unnecessary hospital referrals and potentially enhance some of the diagnostic challenges of acute chest pain in emergency primary care.

## Supplementary Information

**Additional file 1.**

## Data Availability

Data underlying the analyses of this article are presented in the Supplementary Appendix. To preserve patients’ privacy, raw data are not publicly available. Additional data may be shared upon reasonable request to the corresponding author.

## Abbreviations

ACS: Acute coronary syndrome
AMI: Acute myocardial infarction
CHEERS: Consolidated Health Economic Evaluation Reporting Standards statement
DRG: Diagnosis-related groups
ECG: Electrocardiogram
ED: Emergency department
EMS: Emergency medical services
ESC: European Society of Cardiology
GP: General practitioner
hs-cTnT: High-sensitivity cardiac troponin T
ICD-10: International Statistical Classification of Diseases and Related Health Problems, 10th Revision
LOS: Length-of-stay
NSTE-ACS: Non-ST-segment elevation acute coronary syndrome
OAEOC: Oslo Accident and Emergency Outpatient Clinic
OOH: Out-of-hours
OUT-ACS: One-hoUr Troponin in a low-prevalence population of Acute Coronary Syndrome
QALY: Quality-adjusted life year
