# Supplementary material for: Cost-effectiveness of a rule-out algorithm of acute myocardial infarction in low-risk patients: emergency primary care versus hospital setting
Source: BMC Health Serv Res. 2022 Oct 21;22:1274. doi: 10.1186/s12913-022-08697-6 (PMC9587629; doi:10.1186/s12913-022-08697-6)
Supplement: Supplementary file 1 — Additional file 1. [file 12913_2022_8697_MOESM1_ESM.pdf]

Supplementary appendix to the manuscript:

Cost-effectiveness of a rule-out algorithm of acute myocardial infarction in low-risk patients: Emergency primary care versus hospital setting

By

Tonje R. Johannessen, Sigrun Halvorsen, Dan Atar,
John Munkhaugen, Anne Kathrine Nore, Torbjørn Wisløff,
and Odd Martin Vallersnes

Oslo, Norway, 2022

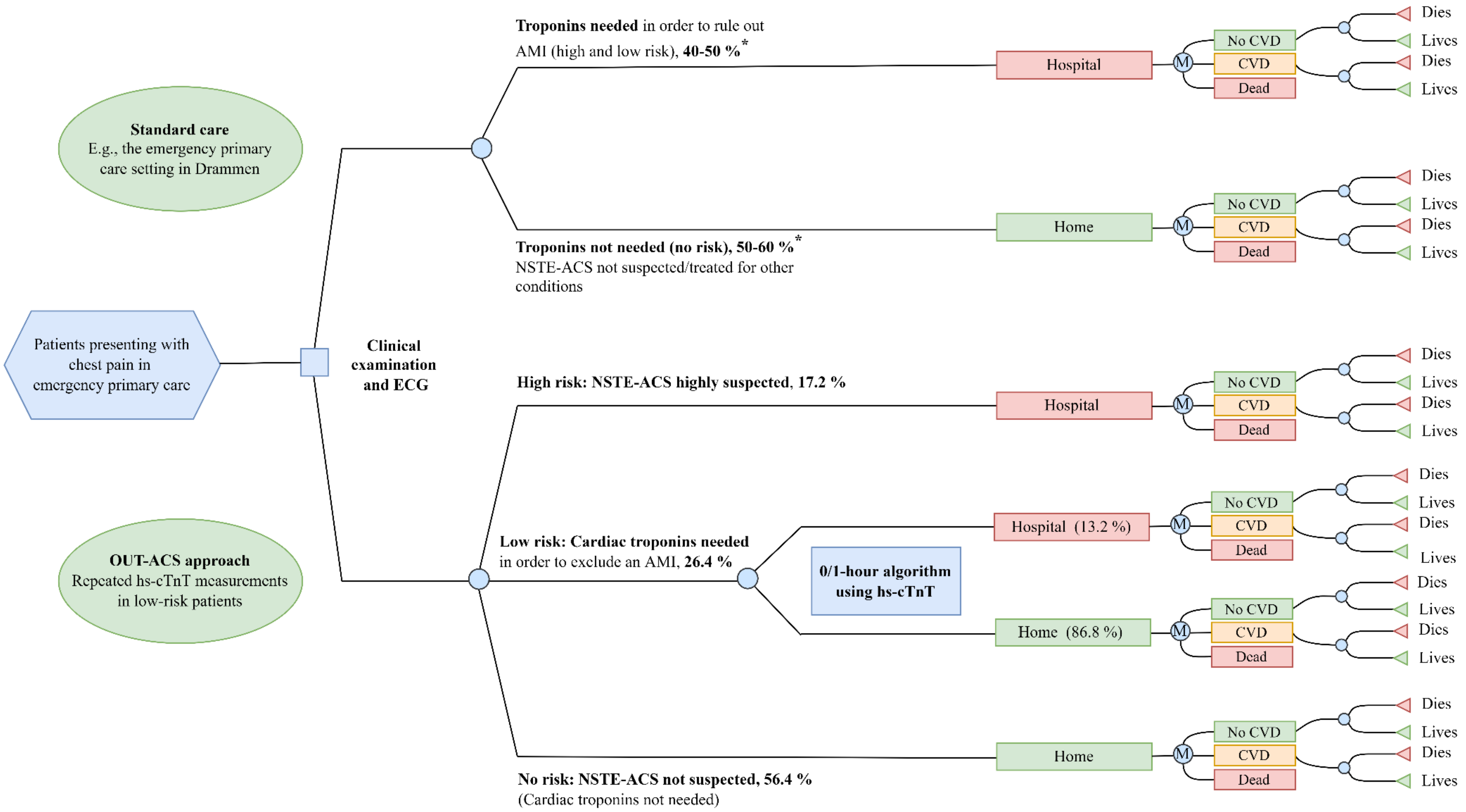

Figure S1 Decision tree

Hexagon indicates patient population, blue quadrant indicates decision made by the treating physcician, blue circles including “M” indicate where the Markov model starts.
* Ref. Burman RA et al., BMC Fam Pract. 2014; Hoorweg BB et al., Heart, 2017; Vester MPM et al., Eur Heart J Qual Care Clin Outcomes, 2020.

AMI: acute myocardial infarction; ECG: electrocardiogram; hs-cTn: high-sensitivity cardiac troponin; NSTE-ACS: acute coronary syndrome; OAEOC: Oslo Accident and Emergency Outpatient Clinic; OUT-ACS: One-hoUr Troponin in a low-prevalence population of Acute Coronary Syndrome

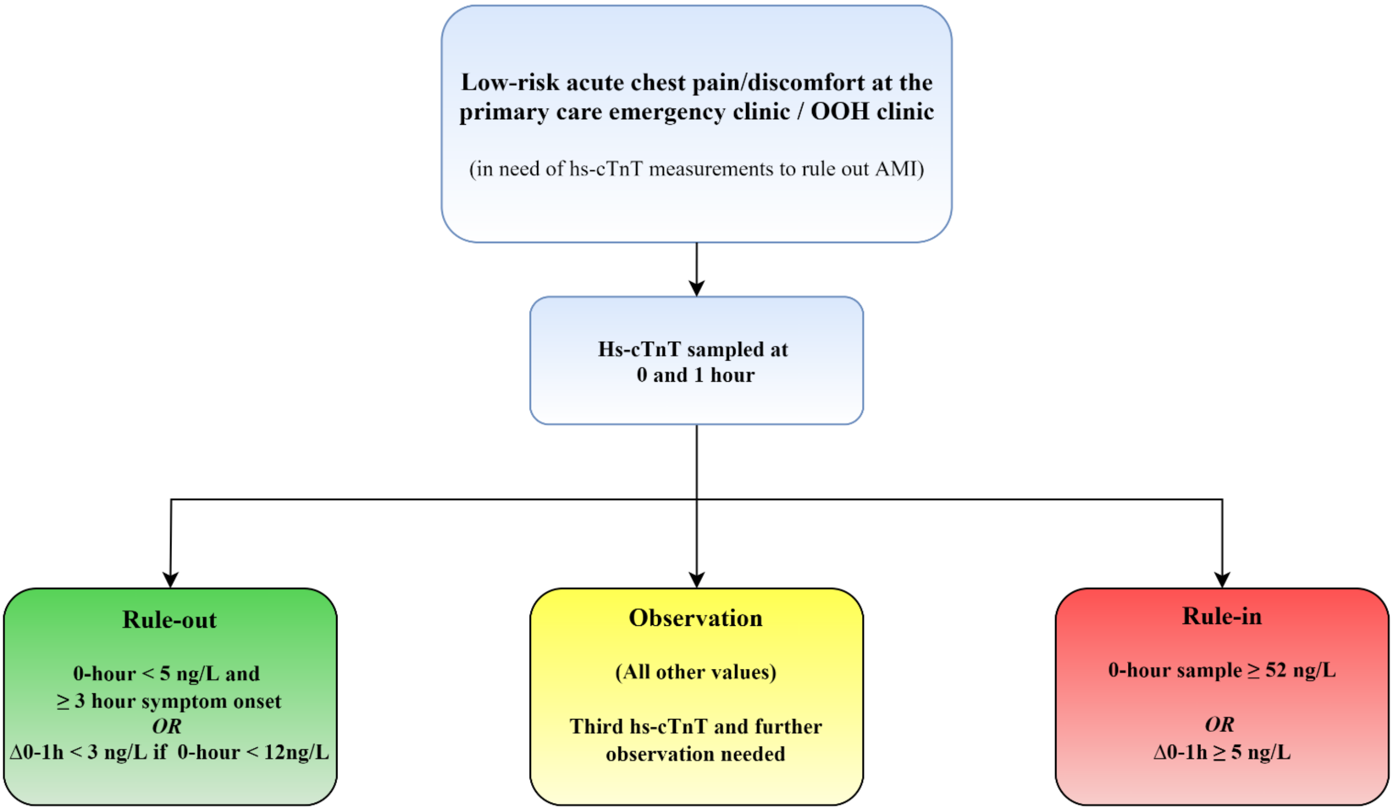

Figure S2 The ESC 0/1-hour algorithm for hs-cTnT

The ESC 0/1-hour algorithm for hs-cTnT presented according to the recent 2020 ESC guidelines on NSTE-ACS.(1) The algorithm uses assay-specific cut-off values and should always be interpreted in conjunction with the clinical assessment and the electrocardiogram.

AMI: acute myocardial infarction; ESC: European Society of Cardiology; hs-cTnT: high-sensitivity cardiac troponin T; ng/L: nanogram per litre; NSTE-ACS: non-ST-segment elevation acute coronary syndrome

Table S1 Estimating health care expenditure

Costs and resources	Estimates and calculations	Total costs (EUR)
**Ambulance transport**<br><br>EUR 559 per transport<br>(2020 figures)	**Transport to primary care emergency clinic:**<br>• 29 % of the low-risk OUT-ACS cohort<br><br>**Transport from primary care emergency to hospital**<br>(recommended for all patients admitted with a suspected NSTE-ACS)<br>• Probability 0.90 (0.75-1.00)	**EUR 162**<br><br><br><br>**EUR 503**
**Primary care emergency clinic, Oslo**<br>(2017 figures)<br><br>Standard costs per patient	**Direct costs per patient**<br>(Including wages triage, doctors, nurses, staff, service, consultation, diagnostics, treatment) = EUR 119<br><br>**Other costs per patient**<br>(including administration, safety, cleaning services, building) = EUR 37<br>Total costs per patient: EUR 157<br><br>Adjusted to 2020 figures:	**EUR 166**
**Additional costs at the primary care emergency clinic**<br><br>Personnel resources and costs (2 models)	**Model 1: Personnel wages** (2020 figures)<br>Additional time spent with the 0/1-hour algorithm x personnel costs per hour:<br><br>**Nurses: 20 min (0.3333)** x<br>• Day: 0.238 x NOK 393 per hour = NOK 93,534<br>• Other: 0.762 x NOK 569 per hour = NOK 433,578<br>Total: NOK 175.70<br><br>**GPs: 50 min (0.8333) x**<br>Registrar (85 %):<br>• Day: 0.238 x NOK 576 per hour x 0.85 = NOK 116,5248<br>• Other: 0.762 x NOK 802 per hour x 0.85 = NOK 519,4554<br>Senior (15 %):<br>• Day: 0.238 x NOK 685 per hour x 0.15 = NOK 24,4545<br>• Other: 0.762 x NOK 940 per hour x 0.15 = NOK 107,442<br>Total: NOK 639.90<br><br>**Total GPs and nurses x 1.3 (**to cover additional social costs) = NOK 1060.28	**Model 1:**<br>**EUR 99**

<table>
<tr><td>Additional diagnostics with the 0/1-hour algorithm
(2020 figures)</td><td>Model 2: Helfo tariffs (2020 figures)

• 4 x 2cd = 4 x 211 x 0.238 = 200,872
• 4 x 2cdd = 4 x 10 x 0.238 x 0.15 = 1,428
• 4 x 2ck = 4 x 174 x 0.762 = 530,352

Total tariffs x 2 (to cover other financing sources):
NOK 732,65 x 2 <u>= NOK 1465,304</u>

Details listed in Table S2</td><td>Model 2:
EUR 137

EUR 41</td></tr>
<tr><td>Additional referrals to supplementary cardiac outpatient testing
(2019 figures)</td><td>• 4.5 % of the non-hospitalised OUT-ACS group
• Assumption: similar referral rate by the regular GP after OAEOC discharge

= Estimated probability 0.10 * CCTA costs NOK 5490

<u>Adjusted to 2020 figures = NOK 5559 * 0.10 = NOK 556</u></td><td>EUR 52</td></tr>
<tr><td>DRG at Drammen hospital

1 DRG = EUR 4269
(2020 figures)

N = 567 patients</td><td>DRG weight x costs per 1 DRG x patients (n):

• DRG 112A: 1.487 x 4269 x 1 = 6348.003
• DRG 143: 0.407 x 4269 x 425 = 738430.275
• DRG 980E: 0.159 x 4269 x 138 = 93670.398
• DRG 981X: 0.173 x 4269 x 3 = 2215.611

= EUR 840 664.287 / 567 patients</td><td>EUR 1483</td></tr>
<tr><td colspan="3">CCTA: coronary computed tomography angiography; DRG: Diagnosis Related Groups; EUR: Euro; GP: general practitioner; NOK: Norwegian Kroner; NSTE-ACS: non-ST-segment elevation acute coronary syndrome; OAEOC: Oslo Accident and Emergency Outpatient Clinic; OUT-ACS: One-hoUr Troponin in a low-risk population of Acute Coronary Syndrome</td></tr>
</table>

Table S2 Additional diagnostic tests at the emergency primary care clinic in Oslo if using the ESC 0/1-hour algorithm

	N	Probability	Price (NOK) 2020 figures	Tariff codes	Unit cost (NOK)*
**Venous blood samples at the clinic**					
Standard blood panel with the 0/1-hour algorithm					
P-hs-cTnT x2	171/171	1.00	33.65 x 2	MB7	134.60
P-CRP	171/171	1.00	9.13	MB3	18.26
P-Creatinine	171/171	1.00	4.94	MB1	9.88
Pt-estimated GFR	171/171	1.00	0.48	MB0	0.96
S-Potassium	171/171	1.00	4.94	MB1	9.88
B-Haemoglobin	171/171	1.00	9.13	MB3	18.26
S-Glucose	171/171	1.00	4.94	MB1	9.88
Additional venous blood samples					
Abdomen panel*	24/171	0.13	4.94 x 6	MB1	7.71
B-leukocytes	70/171	0.41	9.13	MB3	7.49
B-complete blood count	34/171	0.21	33.65	MB7	14.13
P-D-dimer	29/171	0.18	78.95	MB9	28.42
P-NT-proBNP	11/171	0.06	128.28	MB10	15.39
S-Sodium	65/171	0.37	4.94	MB1	3.66
Third hs-cTnT	334/1711	0.20	33.65	MB7	13.46
**Additional diagnostics**					
Additional ECG	335/1711	0.20	95 + 120	10b + 707	86.00
Chest x-ray	15/171	0.09	71 + 250	851 + 899	57.78
**Total costs per low-risk patient**					**NOK 435.76 = EUR 41**

Data on the probabilities of using a specific test or procedure at the OAEOC clinic was extracted from a random selection of the OUT-ACS cohort (10 %, 171/1711). Unit costs are presented in Norwegian Kroner (NOK), where 2020 Euro (EUR) 1.00 = 10.73 NOK. Outpatient radiological and laboratory services were estimated as the reimbursed sum from HELFO plus the patient's fee, multiplied by two, also to include personnel costs at the radiology and lab units.(2) Medical biochemistry (MB) tariffs were provided by The Norwegian Directorate of eHealth.(3)

*Abdomen panel: P-ASAT, P-ALAT, P-GGT, P-ALP, P-bilirubin, P-amylase; each with tariff MB1
CRP: C-reactive protein; ECG: electrocardiogram; ESC: European Society of Cardiology; GFR: glomerular filtration rate; hs-cTnT: high-sensitivity cardiac troponin T; NOK: Norwegian Kroner; NT-proBNP: N-Terminal pro-Brain Natriuretic Peptide

**Table S3 Diagnostic tests and procedures at Drammen Hospital**	
**Diagnostics**	**Probability**
**Venous blood samples at ED admission**	
**Standard blood panel (all patients):**<br>Includes: *B-Sedimentation rate, P-CRP, B-Haemoglobin, B-EVF, Ery-MCV, Ery-MCH, Red cell distribution, B-Leukocytes, B-Neutrophils, B-Lymphocytes, B-Monocytes, B-Eosinophils, B-Basophils, B-thrombocytes, S-Sodium, S-Potassium, P-Calcium, Pt-Estimated GFR, P-Creatinine, P-Cystatin C, P-ALAT, P-ALP, P-Bilirubin, P-Albumin, P-hs-Troponin I x2, B-Glucose, B-HbA1c, P-Cholesterol, P-LDL-cholesterol, P-HDL-cholesterol, P-Triglycerides, Additional serum tube, Additional citrate tube*	**1.00**
P-D-dimer	**0.20**
P-NT-proBNP	**0.30**
**Diagnostics at ED admission**	
ECG	**1.00**
Chest x-ray	**1.00**
Arterial blood gas	**0.07**
**Advanced procedures**	
Stress ECG	0.23 (129/567)
Echocardiogram	0.09 (52/567)
Long-term ECG monitoring	0.01 (7/567)
Holter ECG monitoring	0.003 (2/567)
Other procedures	0.03 (17/567)
**Total**	**0.32 (181/567)** *

The probabilities of diagnostic tests and procedures applied in the hospital assessment were included in the cost-driving estimates of hospital costs (Diagnosis-Related Groups).
* A total of 207 advanced procedures performed in 181 of 567 patients

ECG: electrocardiogram; ED: emergency department; NT-proBNP: N-Terminal pro-Brain Natriuretic Peptide

Table S4 Additional length of stay in the low-risk emergency primary care cohort

	Rule-out<br><br>n = 1232<br>83.0 %	Observation<br><br>n = 243<br>16.4 %	Rule-in<br><br>n = 10<br>0.7 %
0h + 1h sample*	67.1209<br>(SD 9.3652)	67.1209<br>(SD 9.3652)	67.1209<br>(SD 9.3652)
Preparation + lab transport	30 (20-40)	30 (20-40)	30 (20-40)
Central lab	75 (60-90)	75 (60-90)	75 (60-90)
Additional tests for patients in the Observation group (third hs-cTnT, repeated ECG, supplementary tests, lab preparation, transport, and analysis)	-	120<br>(100-140)	-
Discharge by the treating GP	15 (10-20)	15 (10-20)	15 (10-20)
**Total (base case scenario)**	187 min<br>(160-220) =<br>**3.1 hours**	307 min<br>(260-360) =<br>**5.1 hours**	187 min<br>(160-220) =<br>**3.1 hours**
**Total (conservative scenario)**	217 min =<br>**3.6 hours**	357 min =<br>**6.0 hours**	217 min =<br>**3.6 hours**

Estimated additional length of stay among 1485 non-hospitalised patients in the OUT-ACS cohort.
As the ESC 0/1-hour algorithm has not yet been implemented as a clinical routine at the emergency primary care clinic, the estimated additional length of stay is based on best guesses after interviewing senior personnel (GPs and nurses). Brackets illustrate the range of uncertainty in the estimates, where the upper range for each step was chosen in the conservative scenario.

* Time interval (mean) between 0- and 1-hour hs-cTnT samples in the OUT-ACS study, n=1711 patients [(4)]

ECG: electrocardiogram; GP: general practitioner; hs-cTnT: high-sensitivity cardiac troponin T; min: minutes; OAEOC: Oslo Accident and Emergency Outpatient Clinic; OUT-ACS: One-hoUr Troponin in a low-prevalence population of Acute Coronary Syndrome; SD: standard deviation

Additional details concerning the Markov model

The Markov model was created to add a lifetime health perspective. In the model, patients discharged as healthy were calculated separately from those discharged with ACS. For the healthy group, background mortality from Statistics Norway was used.[5] For those with CVD, increased mortality of 1.6 was assumed, as mentioned in the main text. To make adjustments correctly, the rate ratio was multiplied with rates of death and then transformed into a probability using standard methods, as for instance explained by Briggs and colleagues.[6]

Weights for health related quality of life among the general population was based on Norwegian national guidelines for health economic evaluation[2] and from previous Norwegian models,[7] assuming 0.9 as weight for those discharged with CVD. Quality of life weights were added based on the multiplicative principle. As mentioned in the main text of the article, discount rate of 4% were applied and half cycle correction was used to account for events happening on average in the middle of years.

Description of probabilistic sensitivity analysis

We also conducted a probabilistic sensitivity analysis where parameters in our base case model were incorporated as probability distributions. All distributions were incorporated according to common standards, with probabilities as beta or Dirichlet distributions and costs as gamma distributions.[6] Weights for health-related quality of life were incorporated as beta distributions due to weights not likely to be below 0 for any included health states.

Results indicate a 100% probability of the intervention being cost-effective given assumptions in the base case, as all iterations of the Monte Carlo simulation lies in the lower right quadrant of the cost-effectiveness plane (Figure S3). In health economic literature, this is often indicated as being a *dominant* strategy. With two possible strategies, all iterations in the lower right quadrant will also lead to the dominant strategy having a 100% probability of being cost-effective regardless of the variation of the cost-effectiveness threshold (Figure S4).

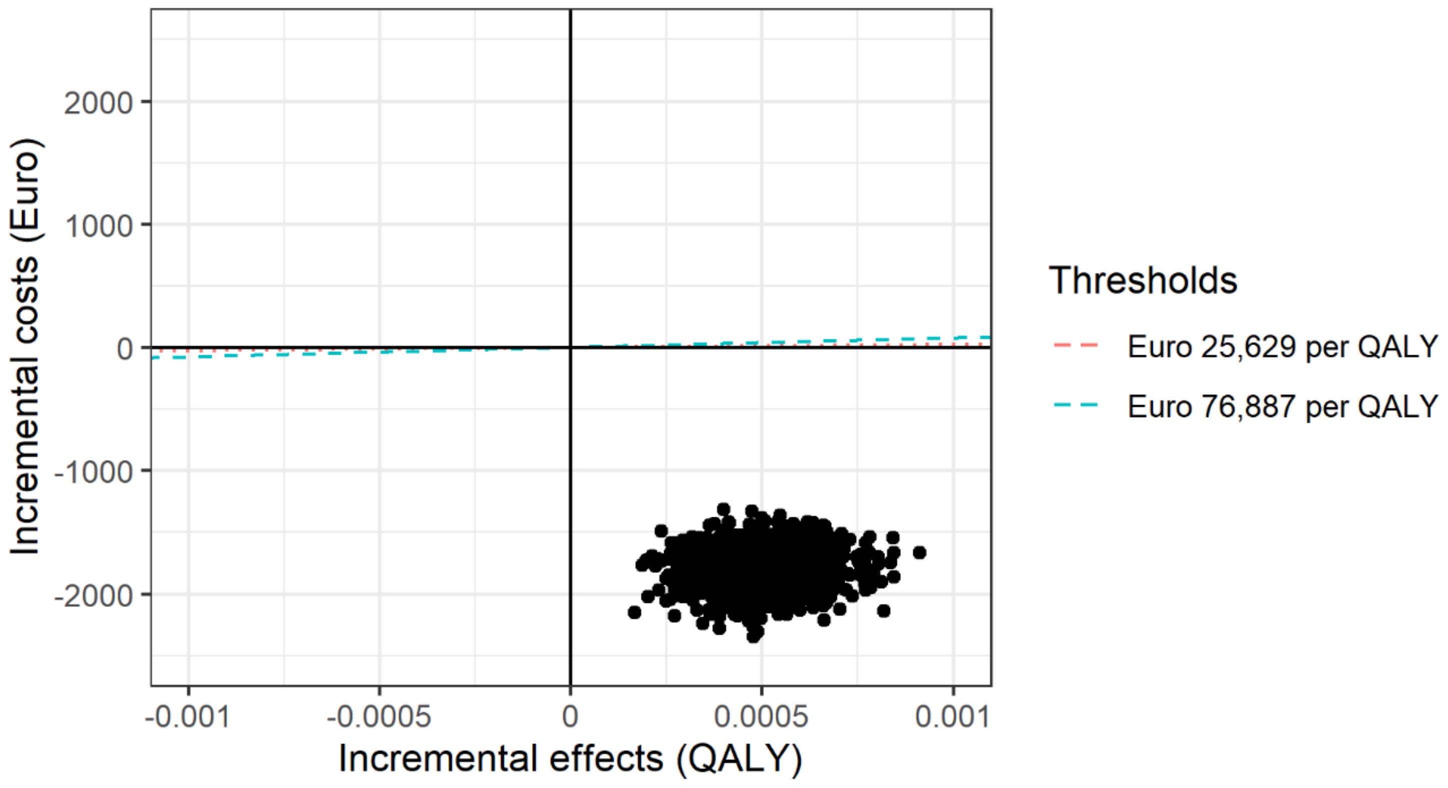

Figure S3 Scatter plot in a cost-effectiveness plane

Dots indicate each iteration from Monte Carlo simulations. Dotted lines for suggested Norwegian thresholds for cost-effectiveness.

QALY: quality-adjusted life-years

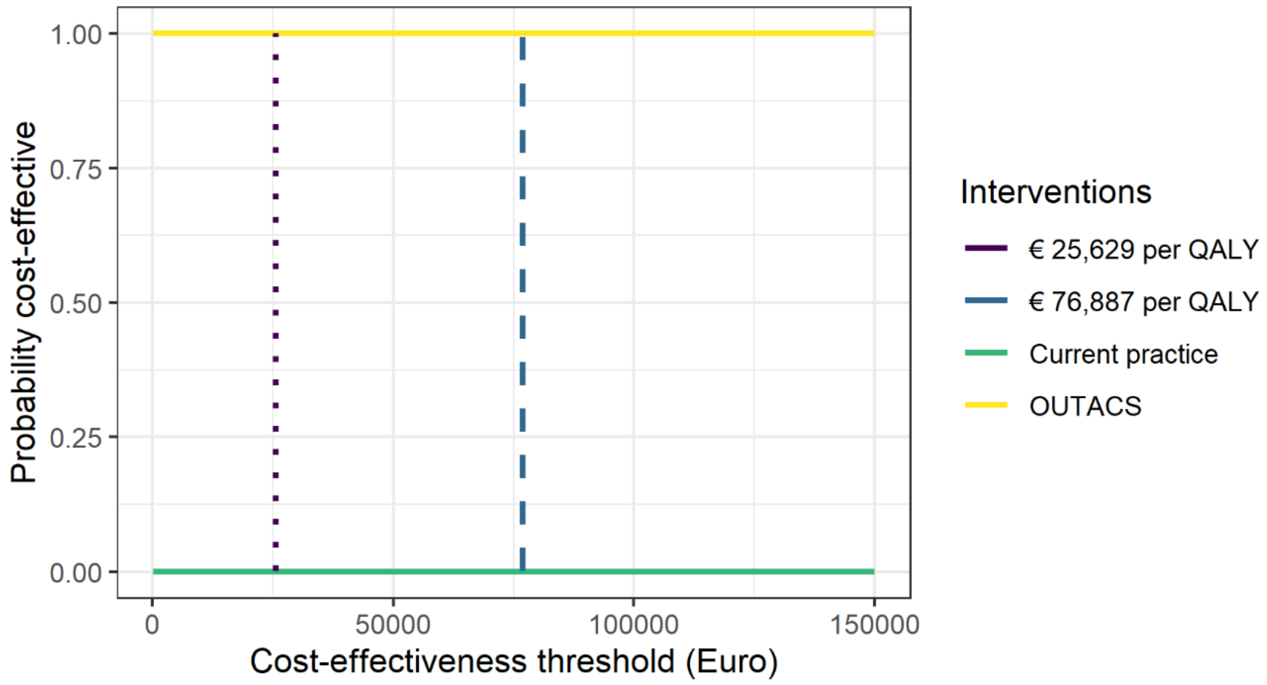

Figure S4 Cost-effectiveness acceptability curve

The curve indicates the probability of cost-effectiveness, given assumptions in the base case scenario.

OUT-ACS: One-hoUr Troponin in a low-prevalence population of Acute Coronary Syndrome; QALY: quality-adjusted life-years

Table S5 Distance from emergency primary care to available hs-cTn assay according to the Norwegian population in 2020

	Norwegian OOH/emergency primary care clinics 2020[8]	Nearest hospital ED with an available hs-cTn assay (24/7)	Kilometres to ED	Minutes to ED (by car)	OOH catchment area[8]	Cumulative population
**1**	Asker og Bærum OOH	Bærum hospital	0	0	127731	127731
**2**	Dalane (Eigersund) OOH	Stavanger University hospital	0	0	24080	151811
**3**	Elverum OOH	Innlandet hospital, Elverum	0	0	36475	188286
**4**	Flekkefjord OOH	Sørlandet hospital, Flekkefjord	0	0	25380	213666
**5**	Frøya OOH	Orkdal hospital	0	0	5151	218817
**6**	Gjøvik OOH	Gjøvik hospital	0	0	65593	284410
**7**	Hammerfest OOH	Hammerfest hospital	0	0	11448	295858
**8**	Kongsberg OOH	Kongsberg hospital	0	0	51224	347082
**9**	Kongsvinger OOH	Kongsvinger hospital	0	0	41468	388550
**10**	Kristiansand OOH	Sørlandet hospital, Kristiansand	0	0	130545	519095
**11**	Kristiansund OOH	Kristiansund hospital	0	0	35621	554716
**12**	Arendal OOH	Arendal hospital	0	0	101248	655964
**13**	Orkdal region OOH	Orkdal hospital	0	0	42001	697965
**14**	Lillehammer OOH	Lillehammer hospital	0	0	43943	741908
**15**	Narvik OOH	University hospital of North Norway, Narvik	0	0	22936	764844
**16**	Nordfjord OOH	Nordfjord hospital	0	0	16587	781431
**17**	Rana OOH	Helgeland hospital, Mo i Rana	0	0	30638	812069
**18**	Ringerike OOH	Ringerike hospital	0	0	65136	877205
**19**	Ryfylke OOH	Stavanger University hospital	0	0	3091	880296
**20**	Skien OOH	Telemark hospital, Skien	0	0	67857	948153
**21**	Sogn LMS OOH	Lærdal hospital	0	0	9100	957253
**22**	Stavanger OOH	Stavanger University hospital	0	0	181948	1139201
**23**	Sunnfjord-Ytre Sogn OOH	Førde Central hospital	0	0	33272	1172473
**24**	Sunnhordland OOH	Stord hospital	0	0	33905	1206378
**25**	Tinn kommunale OOH	Hospitalet Telemark, Skien	0	0	5691	1212069
**26**	Tromsø OOH	University hospital of North Norway, Tromsø	0	0	76974	1289043
**27**	Trondheim, Malvik, Melhus, Midtre-Gauldal OOH	St. Olavs hospital	0	0	242282	1531325
**28**	Ullensvang OOH	Odda Hospital	0	0	11048	1542373
**29**	Vefsn OOH	Helgeland hospital, Mosjøen	0	0	13278	1555651
**30**	Vesterålen OOH	Nordland hospital, Vesterålen Stokmarknes	0	0	30269	1585920
**31**	Voss OOH	Voss hospital	0	0	21703	1607623

	**OOH clinics** *(Table continues)*	**Nearest hospital ED with hs-cTn**	**Kilometres to ED**	**Minutes to ED (car)**	**OOH catchment area**	**Cumulative population**
**32**	Ålesund OOH	Ålesund hospital	0	0	75568	1683191
**33**	Innherred OOH	Levanger hospital	0.45	2	44555	1727746
**34**	Harstad OOH	University hospital of North Norway, Harstad	0.55	2	29576	1757322
**35**	Lofoten OOH	Nordland hospital, Lofoten	0.55	3	13720	1771042
**36**	Drammen OOH	Drammen hospital	0.6	2	128197	1899239
**37**	Hedmarken OOH	Innlandet hospital, Elverum	0.65	2	94875	1994114
**38**	Nord-Østerdal OOH	Innlandet hospital, Tynset	0.9	3	12897	2007011
**39**	Namsos OOH	Namsos hospital	1	3	22396	2029407
**40**	Bodø OOH	Nordland hospital, Bodø	1.2	3	53374	2082781
**41**	Molde OOH	Molde hospital	1.3	3	48755	2131536
**42**	Haugesund OOH	Haugesund hospital	1.5	4	55238	2186774
**43**	Volda, Ørsta OOH	Volda hospital	1.9	4	21298	2208072
**44**	Bergen OOH	Haukeland Universtiy hospital	1.9	5	283929	2492001
**45**	Tønsberg OOH	Vestfold hospital, Tønsberg	2.3	5	107722	2599723
**46**	Notodden OOH	Notodden hospital	3.3	5	25066	2624789
**47**	Oslo OOH	Ullevål hospital	4	12	693494	3318283
**48**	Nedre Romerike OOH	Akershus University hospital	4.4	7	145973	3464256
**49**	Sør-Varanger OOH	Kirkenes hospital	7.3	8	10158	3474414
**50**	Sarpsborg, Rakkestad OOH	Hospitalet i Østfold, Kalnes	7.4	11	64987	3539401
**51**	Porsgrunn OOH	Telemark hospital, Skien	9.8	15	36397	3575798
**52**	Sandnes OOH	Stavanger University hospital	13.3	14	91539	3667337
**53**	Bamble OOH	Telemark hospital, Skien	16.4	23	14061	3681398
**54**	Giske OOH	Ålesund hospital	16.7	18	8462	3689860
**55**	Bråset OOH (Hurum, Røyken)	Drammen hospital	16.9	23	94441	3784301
**56**	Nittedal OOH	Akershus University hospital	17	24	24249	3808550
**57**	Vennesla, Iveland OOH	Sørlandet hospital, Kristiansand	17.1	20	16105	3824655
**58**	Askøy OOH	Haukeland University hospital	17.2	20	29553	3854208
**60**	Sotra OOH	Haukeland University hospital	17.6	23	38316	3892524
**59**	Horten OOH	Vestfold hospital, Tønsberg	18.5	27	27351	3919875
**61**	Fredrikstad, Hvaler OOH	Hospitalet Østfold, Kalnes	18.7	22	87053	4006928
**62**	Sykkylven OOH	Ålesund hospital	20.4	47	12148	4019076
**63**	Klepp, Time OOH	Stavanger University hospital	23.1	24	38504	4057580
**64**	Karmøy OOH	Haugesund hospital	23.3	28	42186	4099766

	OOH clinics *(Table continues)*	Nearest hospital ED with hs-cTn	Kilometres to ED	Minutes to ED (car)	OOH catchment area	Cumulative population
**65**	Gloppen OOH	Nordfjord hospital	25.2	51	5854	4105620
**66**	Helgeland OOH	Helgeland hospital, Sandnessjøen	25.9	60	9741	4115361
**67**	Herøy, Dønna OOH	Helgeland hospital, Sandnessjøen	26.3	63	3148	4118509
**68**	Moss OOH	Østfold hospital, Kalnes	28.7	22	80559	4199068
**69**	Bjørnafjorden, Samnanger OOH	Haraldsplass Diakonale hospital	29.1	38	27393	4226461
**70**	Indre Fosen OOH	St. Olavs hospital	29.8	66	10084	4236545
**71**	Sandefjord OOH	Tønsberg hospital	29.8	25	63764	4300309
**72**	Strand OOH	Stavanger University hospital	30.4	28	12968	4313277
**73**	Nordhordland OOH	Haraldsplass Diakonale hospital	30.7	37	45116	4358393
**74**	Søndre Land OOH	Gjøvik hospital	30.8	31	5617	4364010
**75**	Tysnes OOH	Stord hospital	31.7	79	2869	4366879
**76**	Evenes-Tjeldsund OOH	University hospital of North Norway, Harstad	31.9	34	5564	4372443
**77**	Ørskog OOH	Ålesund hospital	32.5	32	9081	4381524
**78**	Sogndal OOH	Lærdal hospital	33.2	60	11847	4393371
**79**	Hå OOH	Stavanger University hospital	33.6	35	18991	4412362
**80**	Aremark og Halden OOH	Østfold hospital, Kalnes	34	29	32698	4445060
**81**	Værnesregionen OOH	St. Olavs hospital	35	33	31398	4476458
**82**	Follo OOH	Akershus University hospital	36.5	33	126330	4602788
**83**	Jessheim OOH	Akershus University hospital	36.9	28	63508	4666296
**84**	Austevoll OOH	Haukeland University hospital	36.9	85	5236	4671532
**85**	Ulstein-Hareid OOH	Ålesund hospital	37.3	73	13746	4685278
**86**	Kvinnherad OOH	Stord hospital	38.3	88	13071	4698349
**87**	Farsund OOH	Sørlandet hospital, Flekkefjord	40.7	41	9691	4708040
**88**	Larvik OOH	Vestfold hospital, Tønsberg	40.7	34	47204	4755244
**89**	Nesna OOH	Helgeland hospital, Sandnessjøen	42.2	72	1761	4757005
**90**	Grane, Hattfjelldal OOH	Helgeland hospital, Mosjøen	43	40	2779	4759784
**91**	Nes OOH	Akershus University hospital, Kongsvinger	43.1	38	23092	4782876
**92**	Steinkjer OOH	Levanger hospital	43.2	43	26420	4809296
**93**	Indre Østfold OOH	Østfold hospital, Kalnes	43.7	45	52192	4861488
**94**	Lindesnes OOH	Kristiansand hospital	44.3	45	23046	4884534
**95**	Herøy, Sande OOH	Volda hospital	47.4	46	11361	4895895
**96**	HAS OOH	Kristiansund hospital	48	116	11620	4907515
**97**	Indre Namdal OOH	Namsos hospital	48.4	47	5018	4912533

	OOH clinics *(Table continues)*	Nearest hospital ED with hs-cTn	Kilometres to ED	Minutes to ED (car)	OOH catchment area	Cumulative population
**98**	Seljord OOH	Notodden hospital	51.7	50	5291	4917824
**99**	Bremanger OOH	Nordfjord hospital	52.5	75	3629	4921453
**100**	Indre Salten OOH	Nordland hospital, Bodø	53.8	55	16336	4937789
**101**	Røros-Os-Holtålen OOH	Innlandet hospital, Tynset	55.9	53	9453	4947242
**102**	Vågan OOH	Nordland hospital, Vesterålen Stokmarknes	56.3	90	9608	4956850
**103**	Sirdal OOH	Sørlandet hospital	57.3	56	1822	4958672
**104**	Kragerø OOH	Skien hospital	58.5	55	10380	4969052
**105**	Florø OOH	Førde Central hospital	59.4	56	17207	4986259
**106**	Rauma OOH	Molde hospital	59.4	94	7468	4993727
**107**	Lunner-Gran OOH	Gjøvik hospital	59.5	50	22678	5016405
**108**	Luster OOH	Lærdal hospital	60.2	84	5174	5021579
**109**	Eidsvoll OOH	Kongsvinger hospital	62	57	25436	5047015
**110**	Karlsøy OOH	University hospital of North Norway, Tromsø	64.4	65	2200	5049215
**111**	Vanylven OOH	Nordfjord hospital	66	62	3117	5052332
**112**	Fronsvakta OOH	Lillehammer hospital	68.7	58	8842	5061174
**113**	Vega OOH	Helgeland hospital, Sandnessjøen	70.4	118	1200	5062374
**114**	Trysil OOH	Innlandet hospital, Elverum	71.4	58	6627	5069001
**115**	Vik OOH	Voss hospital	73.1	80	2635	5071636
**116**	Aurskog-Høland, Rømskog OOH	Akershus University hospital, Kongsvinger	73.5	65	17390	5089026
**117**	Bardu OOH	University hospital of North Norway, Narvik	74.2	66	16269	5105295
**118**	Kvam OOH	Voss hospital	75.5	80	8457	5113752
**119**	Lyngen OOH	University hospital of North Norway, Tromsø	79.2	107	2794	5116546
**120**	Vikna, Nærøy OOH	Namsos hospital	87.6	113	9623	5126169
**121**	Etne, Vindafjord OOH	Stord hospital	89.5	85	12776	5138945
**122**	Gildeskål OOH	Nordland hospital, Bodø	90.7	82	1950	5140895
**123**	Balsfjord-Storfjord OOH	University hospital of North Norway, Tromsø	92.2	78	7388	5148283
**124**	Brønnøy OOH	Helgeland hospital, Sandnessjøen	92.4	163	9892	5158175
**125**	Nore og Uvdal OOH	Kongsberg hospital	93.7	85	2439	5160614
**126**	Værøy OOH	Nordland hospital, Lofoten	96.2	175	728	5161342
**127**	Valdres OOH	Gjøvik hospital	97.3	88	17578	5178920
**128**	Fosen OOH	St. Olavs hospital	101	125	14611	5193531
**129**	Oppdal OOH	Innlandet hospital, Tynset	102	86	7001	5200532
**130**	Sunndal OOH	Kristiansund hospital	103	99	7036	5207568

	OOH clinics *(Table continues)*	Nearest hospital ED with hs-cTn	Kilometres to ED	Minutes to ED (car)	OOH catchment area	Cumulative population
**131**	Nord-Gudbrandsdal OOH	Lillehammer hospital	109	90	18262	5225830
**132**	Sauda OOH	Haugesund hospital	112	116	4595	5230425
**133**	Suldal OOH	Haugesund hospital	114	116	3804	5234229
**134**	Gol-Hemsedal OOH	Ringerike hospital	116	98	7094	5241323
**135**	Meløy OOH	Nordland hospital, Bodø	117	103	6288	5247611
**136**	Engerdal OOH, Trysil	Innlandet hospital, Tynset	119	98	1268	5248879
**137**	Vevelstad OOH	Helgeland hospital, Mosjøen	119	153	462	5249341
**138**	Lurøy OOH	Helgeland hospital, Sandnessjøen	122	183	1890	5251231
**139**	Bindal OOH	Hospitalet Namsos	127	56	1426	5252657
**140**	Tokke-Vinje OOH	Telemark hospital, Skien	128	124	5877	5258534
**141**	Solund OOH	Førde Central hospital	129	200	802	5259336
**142**	Stor-Elvdal OOH	Lillehammer hospital	138	128	2419	5261755
**143**	Rødøy OOH	Helgeland hospital, Mo i Rana	140	181	1213	5262968
**144**	Tana-Nesseby OOH	Kirkenes hospital	140	118	3844	5266812
**145**	Øvre Hallingdal OOH	Ringerike hospital	140	119	7947	5274759
**146**	Alta OOH	Hammerfest hospital	142	134	20789	5295548
**147**	Porsanger OOH	Hammerfest hospital	143	129	3998	5299546
**148**	Træna OOH	Helgeland hospital, Mo i Rana	143	319	435	5299981
**149**	Leka OOH	Namsos hospital	151	166	557	5300538
**150**	Finnsnes OOH	University hospital of North Norway, Tromsø	159	133	18315	5318853
**151**	Hol OOH	Ringerike hospital	159	135	4441	5323294
**152**	Måsøy OOH	Hammerfest hospital	168	153	1225	5324519
**153**	Vadsø OOH	Kirkenes hospital	171	144	5788	5330307
**154**	Kåfjord OOH	University hospital of North Norway, Tromsø	179	150	2071	5332378
**155**	Nordkapp OOH	Hammerfest hospital	181	167	3162	5335540
**156**	Bykle-Valle OOH, Hovden	Telemark hospital, Skien	194	184	2129	5337669
**157**	Steigen OOH	Bodø hospital	212	183	5374	5343043
**158**	Karasjok OOH	Hammerfest hospital	218	186	2628	5345671
**159**	Nordreisa OOH	University hospital of North Norway, Tromsø	224	199	4861	5350532
**160**	Kvænangen OOH	Hammerfest hospital	229	226	1191	5351723
**161**	Båtsfjord OOH	Kirkenes hospital	244	193	2221	5353944
**162**	Skjervøy OOH	University hospital of North Norway, Tromsø	245	217	2927	5356871
**163**	Vardø OOH	Kirkenes hospital	245	206	2029	5358900
**164**	Kautokeino OOH	Hammerfest hospital	271	239	2910	5361810
**165**	Berlevåg OOH	Kirkenes hospital	272	216	957	5362767
**166**	Hasvik OOH	Hammerfest hospital	278	318	1005	5363772

	OOH clinics *(Table continues)*	Nearest hospital ED with hs-cTn	Kilometres to ED	Minutes to ED (car)	OOH catchment area	Cumulative population
**167**	Nordkyn OOH	Kirkenes hospital	336	293	2422	5366194
**168**	Øksfjord OOH	University hospital of North Norway, Tromsø	355	320	888	5367082
**169**	Røst OOH	Nordland hospital, Bodø	?	?	498	5367580

The list of Norwegian OOH clinics in 2020 and catchment areas was obtained from the Norwegian Research Centre (NORCE).[(8)]
Distance and minutes by car to nearest hospital ED with available hs-cTn assay was investigated for the purpose of this study by contacting each hospital by phone and Google Maps.

ED: emergency department; hs-cTn: high-sensitivity cardiac troponin; OOH: out-of-hours

References

1. Collet JP, Thiele H, Barbato E, Barthelemy O, Bauersachs J, Bhatt DL, et al. 2020 ESC Guidelines for the management of acute coronary syndromes in patients presenting without persistent ST-segment elevation. Eur Heart J. 2021;42(14):1289-367.
2. The Norwegian Medicines Agency. Guidelines for the submission of documentation for single technology assessment (STA) of pharmaceuticals [Statens legemiddelverk]. 2020 [cited 2021 June 03]. Available from: https://legemiddelverket.no/english/public-funding-and-pricing/documentation-for-sta/guidelines-for-the-submission-of-documentation-for-single-technology-assessment-sta-of-pharmaceuticals.
3. The Norwegian Directorate of eHealth. [Norsk laboratoriekodeverk] [Lab koder]. Norwegian Ministry of Health and Care Services; 2020 [cited 2021 September 10]. Available from: https://www.ehelse.no/kodeverk/laboratoriekodeverket.
4. Johannessen TR, Vallersnes OM, Halvorsen S, Larstorp ACK, Mdala I, Atar D. Pre-hospital One-Hour Troponin in a Low-Prevalence Population of Acute Coronary Syndrome: OUT-ACS study. Open Heart. 2020;7(2).
5. Norway S. Table 07902: Life tables, by sex and age 1966 - 2021 [Internet] 2022 [cited 2022 13 August]. Available from: https://www.ssb.no/en/statbank/table/07902/.
6. Briggs A, Claxton K, Sculpher M. Decision Modelling for Health Economic Evaluation. 1 ed: Oxford University Press; 2006.
7. Wisløff T, Hagen G, Klemp M. Economic evaluation of warfarin, dabigatran, rivaroxaban, and apixaban for stroke prevention in atrial fibrillation. Pharmacoeconomics. 2014;32(6):601-12.
8. Allertsen M, Morken T. 2021, Organisation of Emergency Primary Health Care in Norway 2020, Norwegian Research Centre (NORCE), https://norceresearch.brage.unit.no/norceresearch-xmlui/bitstream/handle/11250/2755945/Offisiell%20oversikt%20legevakter%20og%20legevaktsentraler%20i%20Norge%202020.xlsx?sequence=6&isAllowed=y.
